# Expression of the Cavin Family in Childhood Leukemia and Its Implications in Subtype Diagnosis and Prognosis Evaluation

**DOI:** 10.3389/fped.2022.815421

**Published:** 2022-06-03

**Authors:** Jing Yang, Junbin Huang, Huabin Wang, Yong Liu, Yanlai Tang, Chao Lin, Qin Zhou, Chun Chen

**Affiliations:** ^1^Department of Pediatrics, The Seventh Affiliated Hospital, Sun Yat-sen University, Shenzhen, China; ^2^Department of Pediatrics, The First Affiliated Hospital, Sun Yat-sen University, Guangzhou, China

**Keywords:** Cavin family, childhood leukemia, diagnosis, prognosis, implications

## Abstract

**Background:**

Caveolae are plasma membrane subdomains of many mammalian cells that play critical roles in cellular processes, including endocytosis, signal transduction and tumorigenesis. Cavin proteins are essential for caveola formation, structure and function and are reported to be involved in various human diseases, but little is known about their expression and prognostic value in leukemia.

**Methods:**

We performed a detailed analysis of Cavin family mRNA expression levels in different cancer tissues vs. normal tissues via the ONCOMINE, Gene Expression Profiling Interactive Analysis (GEPIA) and Cancer Cell Line Encyclopedia (CCLE) databases. Then, we used qRT-PCR and Western blotting to validate Cavin1-4 expression in 10 fresh leukemia samples. Moreover, we estimated their prognostic value in leukemia with the R programming language and GEPIA database.

**Results:**

The expression of Cavin members is low in most human cancers, especially in leukemia. Cavin-1 and Cavin-2 are often more expressed in myeloid leukemia than lymphoblastic leukemia, but Cavin-4 has the opposite pattern. Interestingly, low expression of CAVIN1 and CAVIN4 is correlated with poorer outcome but low CAVIN2 expression is associated with a significantly better leukemia prognosis in leukemia.

**Conclusion:**

The Cavin family showed significant expression differences between leukemia and normal cells. High Cavin-2 and low Cavin-4 levels predict poor survival and could be promising subtype diagnosis and prognosis biomarkers for leukemia.

## Background

Leukemias are a group of hematologic disorders characterized by the aberrant proliferation and include acute lymphoblastic leukemia (ALL), acute myeloid leukemia (AML), chronic lymphocytic leukemia (CLL), and chronic myeloid leukemia (CML) (1). Acute leukemia develops with a peak incidence between 1 year and 4 years but chronic leukemia is very rare in children (2). Different types of leukemia have different treatment strategies and prognosis. Identifying the potential molecular biomarkers of subtyping, prognostic in leukemia is important for selecting the best treatment strategy and improving outcomes.

Caveolae or “little cave” is a cave-shaped invagination structure of the cell plasma membrane subdomains measuring 50–100 nm in many mammalian cells. Caveolae consists of caveolin and Cavin proteins and involved in endocytosis, cholesterol homeostasis, signal transduction and tumorigenesis (3). The Cavin family of proteins is essential for caveola formation, shape, size, structure, and functions (4). The Cavin family Consists of four Cavin proteins termed Cavin1~4 and is encoded by the polymerase 1 transcript release factor (PTRF, CAVIN1), serum deprivation response protein (SDPR, CAVIN2), protein kinase c delta binding protein (PRKCDBP, CAVIN3), and muscle-related skeletal and cardiac (MURC, CAVIN4) genes. Cavin-1 was found to mainly facilitate the number of caveolae and Cavin-2 is reported to contribute to the caveolae morphology repair (5). Cavin-3 is involved in the formation of caveolae, which is affected by its LZ domain and the Caveolin-1 expression (6). Cavin-4 is a muscle-specific component of the Cavin complex and able to interact with Cavin-2, which is associated with the sarcolemmal caveolae complexes (7).

Caveolae is associated with tumor growth, invasion, metastasis, multidrug resistance and angiogenesis in cancer (8). Cavin family members are major players in caveola biology. Recently, some studies have pointed to a potential role for Cavin family members in breast cancer, lung cancer and AML (6, 9). In the majority of human cancers, Cavin-1 is downregulated along with Cavin-2, Cavin-3 and Cavin-4 compared in cancer tissues vs. control tissues (9). Cavin-1 was found to involved in cell adhesion, senescence, cell metastasis and drug resistance (10). The role of Cavin-1 in cancer is controversial as it has both tumor suppression and promotion activities in different cancers or the same type of cancer at different stages of disease progression (6). Some observations point to Cavin-1 promoting tumor migration in pancreatic cancer cells by cooperating with caveolin-1 but inhibiting invasiveness and metastasis by matrix metalloproteinase 9 (MMP-9) production, neutralizing CAV1 tumor-promoting properties in PC3 pancreatic cancer cells (11, 12). Cavin-1 was reported to enhance resistance to anticancer treatment in colorectal cancer (13) but reduce the drug resistance in prostate cancer cells (14). Cavin-2 is a membrane-bound phosphatidylserine-binding protein and a substrate for Protein Kinase C (PKC), which is a critical organizer of caveolae and regulator of angiogenesis (15, 16). Cavin-2 recruits Cavin-1 and Caveolin-1 protein to the plasma membrane inducing drug resistance by facilitating the formation of lipid rafts in MDR cell lines (12, 14). Cavin-3 suppresses tumorigenic properties, interacts with the DNA damage repair pathway, regulates cancer cell invasion or metastasis and induces drug resistance by decreasing cell sensitivity to oxaliplatin (12). The absence or downregulation of Cavin-3 with promoter hypermethylation was found in various types of human cancer, especially at late stages of cancer, which could predict a low progression-free survival rate (17, 18). There are few reports about Cavin-4 in cancers. Their roles in leukemia need further investigation and clarification.

To investigate the relationship between dysregulation of Cavins levels and the classification and prognosis of children with leukemia. In the present study, we performed a comprehensive bioinformatics analysis to evaluated the relationship of the Cavin family with clinicopathological features and patient survival in leukemia by dataset analysis. We detected the expression levels of Cavin family members in 10 children with leukemia by qRT-PCR and Western blotting. These results for leukemia typing and prediction of prognosis in children with leukemia by targeting caveolae-related genes.

## Materials and Methods

### Patients and Samples

Data for a total of 10 patients with childhood leukemia and 6 matched healthy donors providing peripheral blood or bone marrow were obtained from The First and Seventh Affiliated Hospital of Sun Yat-sen University from 2020 to 2021. Clinical features such as age, sex, diagnosis date, disease state and white blood cell count were obtained from hospital records (Table 1).

**Table 1:** The patients clinical information.

**Patient number**	**Gender/Age (yr)**	**Diagnosis**	**Sample**	**Initial or relapsed disease**	**WBC count (10^**9**^/L)**
1	M/1	ALL-L2, B	PB	Initial	31.03
2	F/6	ALL-L2, B	PB	Relapsed	53.02
3	F/5	ALL-L2, T	PB	Relapsed	34.78
4	F/10	ALL-L2, B	PB+ BM	Initial	31.01
5	F/4	ALL-L2, B	PB	Initial	101.12
6	F/6	ALL-L2, T	PB	Initial	19.21
7	M/14	ALL-L2, B	PB+BM	Relapsed	13.42
8	M/6	AML	PB	Initial	10.31
9	F/5	AML	PB	Initial	39.02
10	M/10	AML	PB	Initial	97.03

Mononuclear cells were isolated by Histopaque gradient centrifugation (density 1.077; Sigma-Aldrich, Shanghai). Contaminating red blood cells were removed by incubation in 0.8% ammonium chloride solution for 10 min. After washing, the cells were suspended in IMDM supplemented with 10% FBS.

### RNA Extraction and Quantitative Real-Time PCR

All the cells were collected and stored in liquid nitrogen. Total RNA was isolated using RN001 (Yishan Biotechnology, Shanghai, China) and reverse transcribed into cDNA by the cDNA Synthesis Kit RT001 (Yishan Biotechnology, Shanghai, China). qPCR was performed using the SYBR Premix Ex Taq Kit (Takara, Dalian, China) according to the manufacturer's recommended protocol. The reaction procedure was 95°C for 30 s, 95°C for 5 s and 60°C for 30 s in 40 cycles. The primers for the Cavin family members were as follows: Cavin-1, 5′-GGGCCGTAGACCAGATCCA-3′ and 5′-CTTGCTCACCGTATTGCTCGT-3′; Cavin-2, 5′- CATCCGGGACAACTCACAGG-3′ and 5′-CAGCGTCTAGCATGTTCACCA-3′; Cavin-3, 5′-CACGTTCTGCTCTTCAAGGAG-3′ and 5′-TGTACCTTCTGCAATCCGGTG-3′; and Cavin-4, 5′-TAAAATCCGTCCAGATTGACCTG-3′ and 5′-GAGCACTAACTTTTCGGGTTTTC-3′. The primer sequences for GAPDH were 5′-CCCCGCTACTCCTCCTCCTAAG-3′ and 5′-TCCACGACCAGTTGTCCATTCC-3′. Relative mRNA expression was analyzed by the 2–ΔCq method.

### Western Blotting Analysis

The cells were collected and lysed in radioimmunoprecipitation assay (RIPA) buffer (1 × PBS, 1% NP-40, 0.5% sodium deoxycholate, 0.1% SDS, 0.1 mg/ml phenyl-methanesulfonyl fluoride, 20 mM sodium fluoride, 0.2 mM sodium orthovanadate, and Complete Protease Inhibitor Mix, one tablet per 50 ml). The protein concentration of each sample was quantified by BCA assay, and 30 μg of protein was separated by 12% SDS-PAGE and transferred to nitrocellulose membranes. The membranes were blocked with 5% non-fat milk for 1 h and then incubated with anti-Cavin-1 (1:1,000, Cell Signaling Tech, Beverly, MA), anti-Cavin-2 (1:500, Abcam Corp, USA) and anti-actin antibodies (1:8000, Sigma-Aldrich, Shanghai, China) overnight at 4°C. The membranes were washed and incubated with secondary antibodies for 1 h. The membranes were scanned by using an Odyssey infrared imaging system (LI-COR).

### ONCOMINE Analysis

ONCOMINE (https://www.oncomine.org/), an open online cancer microarray database, was used to analyze the transcript levels of the Cavin family in different cancers. The mRNA expression levels of the Cavin family members in clinical cancer specimens were compared with those in normal controls using Student's *t*-test to generate the *p*-value.

### GEPIA Database

Gene Expression Profiling Interactive Analysis (GEPIA) is a newly developed interactive web server for analyzing the RNA sequencing expression data of 715 gene expression datasets of 9,736 tumor and normal samples from The Cancer Genome Atlas (TCGA), Gene Expression Omnibus (GEO) and Genotype-Tissue Expression (GTEx) projects using a standard processing website (http://gepia.cancer-pku.cn/). GEPIA can be applied for tumor vs. normal differential expression analysis, detailed analyses according to cancer types or pathological stages, patient survival analysis, correlation analysis, multiple gene comparison and dimensionality reduction analysis (19).

### BloodSpot Analysis

BloodSpot (http://servers.binf.ku.dk/bloodspot/) is an open online malignant haematopoiesis database of gene expression profiles from FACS sorted healthy and malignant haematopoietic cells. It was used to analyze the mRNA expression of the Cavin family in different types of leukemia compared with those in healthy bone marrow using oligonucleotide microarray chips. Datasets are organized by organism of origin and disease status, and includes human healthy hematopoietic cells, human leukemia and healthy mouse hematopoietic cells.

### LinkedOmics Database

LinkedOmics (http://www.linkedomics.orglogin.php) is a new tool in the software ecosystem for disseminating data from large-scale cancer omics projects. To reduce redundant efforts, the LinkedOmics database uses preprocessed and normalized data from the Broad TCGA Firehose and Clinical Proteomic Tumor Analysis (CPTAC) data portals and focuses on the discovery and interpretation of attribute associations, thus complementing existing cancer data portals (20).

### Cancer Cell Line Encyclopedia Database

The CCLE (https://www.broadinstitute.org/ccle) project is a collaboration between the Broad Institute, the Novartis Institutes for Biomedical Research and the Genomics Institute of the Novartis Research Foundation that can be used to conduct a detailed genetic and pharmacologic characterization of a large panel of human cancer models, develop integrated computational analyses that link distinct pharmacologic vulnerabilities to genomic patterns and translate cell line integrative genomics data into cancer patient stratification systems (21). The CCLE provides public access to genomic data analysis and visualization tools for ~1,000 kinds of cell lines. The expression of the Cavin family in cancer cell lines was verified with the CCLE database.

### Statistical Analysis

The statistical results were recorded as the mean ± 95% confidence intervals unless otherwise stated and analyzed using GraphPad Prism 8.0 (San Diego, CA). The non-parametric Mann-Whitney U-test was used to compare the differences between two groups. The correlation between Cavin family member expression and clinical characteristics was analyzed by the chi-square test (χ^2^). Survival analysis was evaluated using the Kaplan-Meier method and Cox proportional hazards model. Differences with *p* < 0.05 were considered statistically significant.

## Results

### Cavin Family Members Are Expressed at Low Levels in Lymphoblastic Leukemia

**Figure 1:**
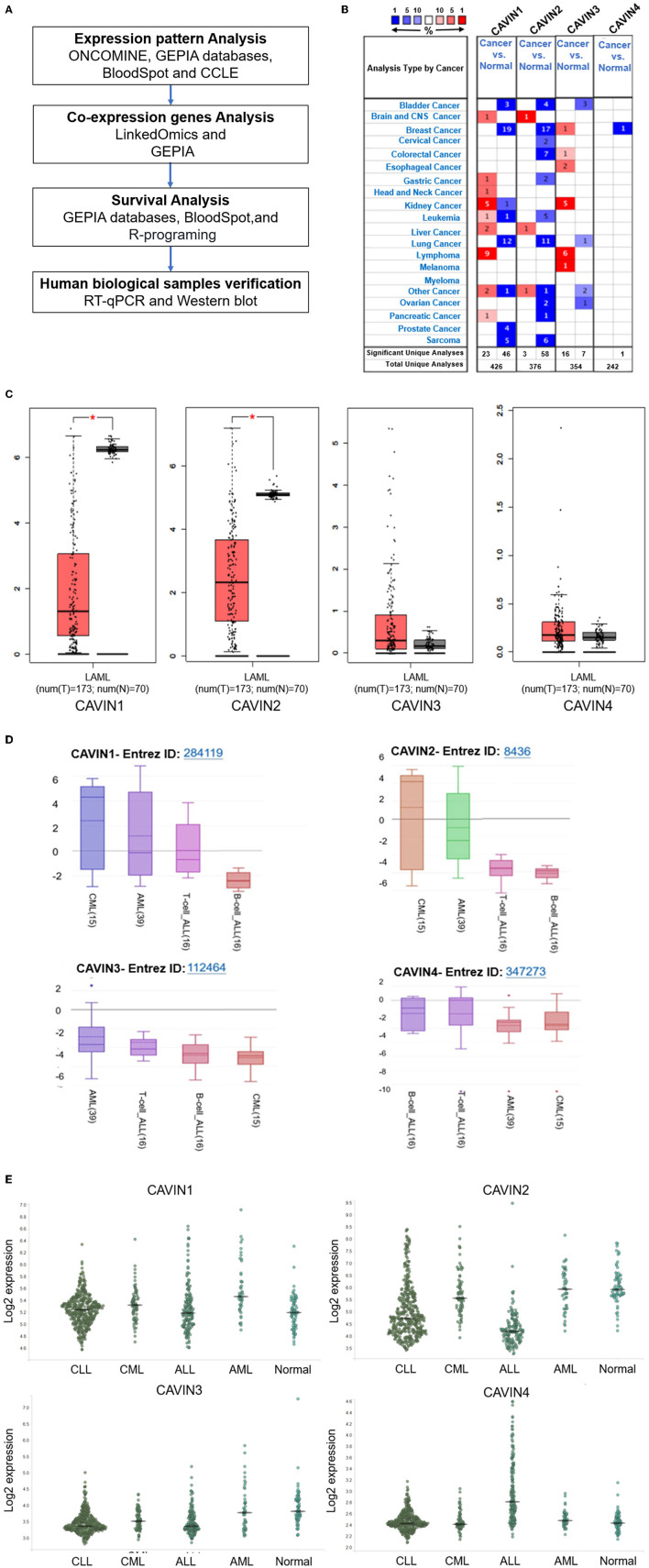
The expression of the Cavin family. **(A)** The workflow diagram of this study. **(B)** The transcription levels of CAVINs in different types of cancers discovered by ONCOMINE. The expression of Cavin members is under-expressed in most human cancers. The mRNA expression of CAVIN1 and CAVIN2 was significantly lower in human leukemia than that in normal cells. **(C)** The mRNA expression levels of Cavin family members in Acute Myeloid Leukemia (AML) detected by GEPIA. **(D,E)** The expression of CAVIN1-4 in different type of Leukemia Cell Lines analyzed by CCLE **(D)** and BloodSpot **(E)**. CAVIN1 and CAVIN2 are higher in myeloid leukemia, while CAVIN4 expression is just the opposite in leukemia. LAML, Acute Myeloid Leukemia; CML, Chronic Myeloid Leukemia; T-ALL, T-cell Acute Lymphoblastic Leukemia; B-ALL, B-cell Acute Lymphoblastic Leukemia; CAVIN1 and CAVIN2 are often more expressed in myeloid leukemia than lymphoblastic leukemia, but CAVIN4 has the opposite pattern.

As shown in Figure 1B, the mRNA expression levels of Cavin family members are downregulated in most human cancers, especially in leukemia analyzed by ONCOMINE database. We further confirmed this result with the GEPIA databases (http://gepia.cancer-pku.cn/) and found that all Cavin family members are downregulated in leukemia, significantly CAVIN1 and CAVIN2 (Figure 1C). We next furtherly expanded the process of detailed annotation of preclinical human leukemia models by assessing the CCLE (https://www.broadinstitute.org/ccle) and BloodSpot (http://servers.binf.ku.dk/bloodspot/) database. We found that the expression level of both CAVIN1 and CAVIN2 in myeloid leukemia is higher than that in lymphocytic leukemia, while the expression of CAVIN4 is higher in lymphocytic leukemia. There is no difference in the expression of CAVIN3 between the two types of leukemia (Figures 1D,E).

### The Correlations Between Cavin Family Members in Leukemia

**Figure 2:**
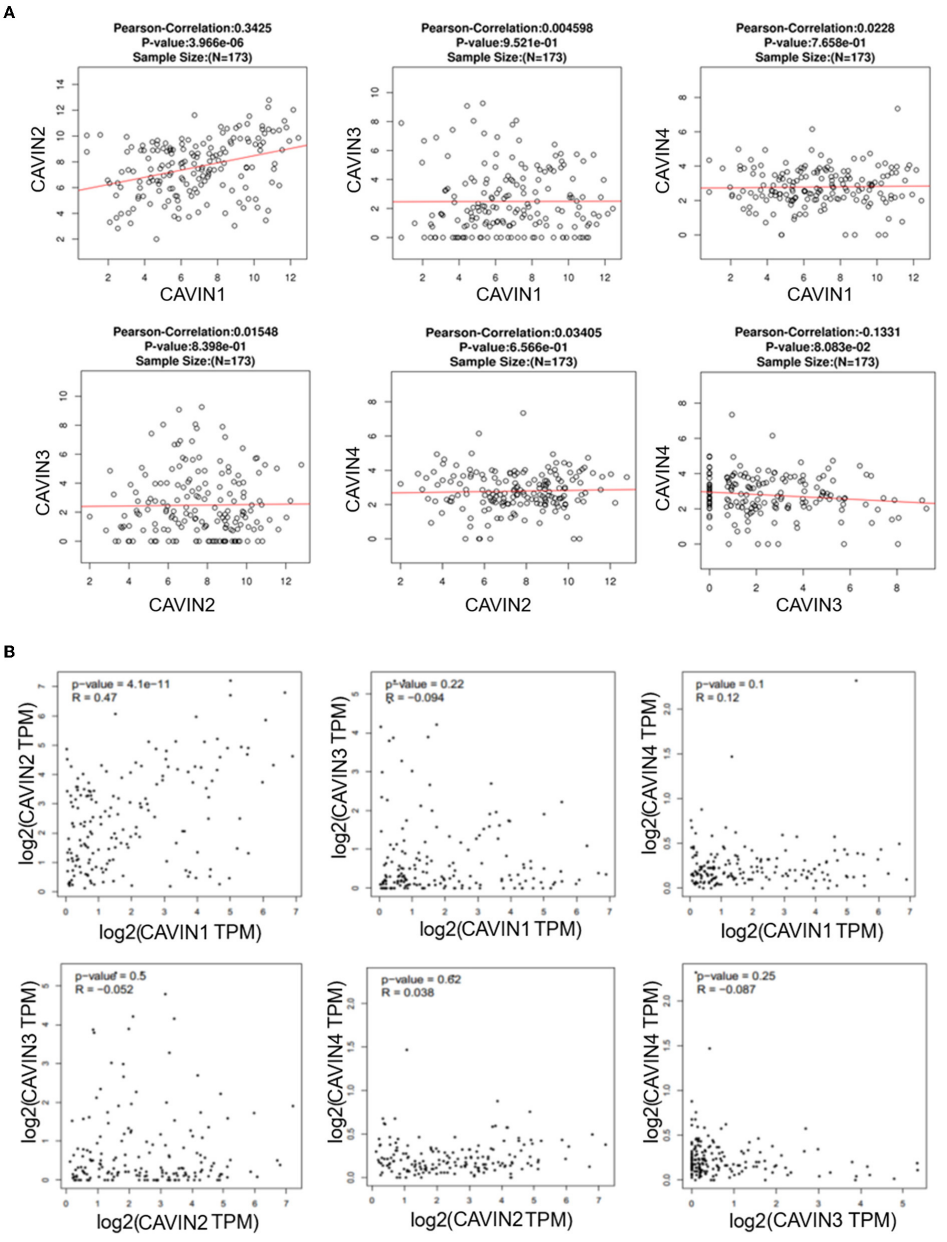
The correlation between Cavin family member pairs in Leukemia by LinkedOmics **(A)** and GEPIA **(B)**. CAVIN1 is linearly correlated with CAVIN2, and there is no significant correlation between the remaining Cavins members.

Next, we analyzed pairwise correlations among CAVIN1, CAVIN2, CAVIN3, and CAVIN4 *via* the LinkedOmics database and found that CAVIN1 was predominantly correlated with CAVIN2 (R = 0.3425, *p* < 0.05), but there was no significant correlation between other members (Figure 2A). Furthermore, we verified the results using the GEPIA dataset. Consistently, the mRNA level of CAVIN1 was positively correlated with CAVIN2 (R = 0.47, *p* < 0.05) but not significantly correlated with any other Cavin family members in leukemia (Figure 2B).

### Cavin Family Expression Predicts the Prognosis of Leukemia

**Figure 3:**
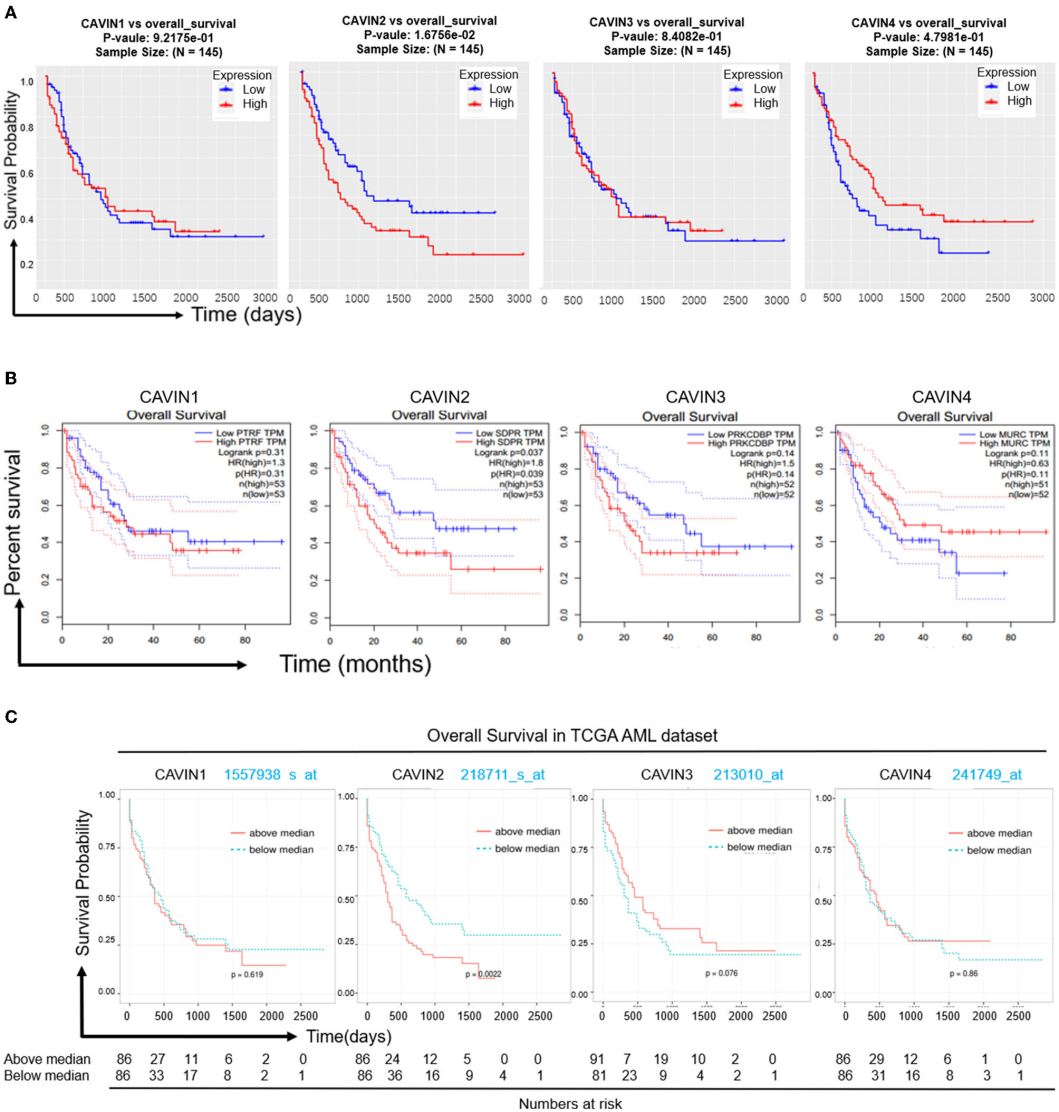
The Prognostic value of mRNA level of Cavin family in leukemia patients. **(A)** Data from LinkedOmics was evaluated using the Kaplan-Meier method and Cox's proportional hazards model by R-programing. **(B)** The prognostic value of mRNA level of Cavin family in Leukemia patients, analyzed by GEPIA. Blue line indicates low expression, red line indicates high expression. **(C)** The overall survival of mRNA level of Cavin family in AML cell lines, analyzed by BloodSpot database. Green line indicates low expression, red line indicates high expression. *p* < 0.05 was considered as statistically significant.

Finally, we investigated the prognostic value of CAVIN1, CAVIN2, CAVIN3, and CAVIN4 in leukemia using the R programming language to assess data from LinkedOmics. In particular, higher CAVIN4 expression was significantly associated with better OS in leukemia (*p* = 0.048). However, patients with CAVIN2-overexpressing leukemia had even better survival (*p* = 0.017). There were no significant differences in either high or low levels of CAVIN1 and CAVIN3 in the leukemia prognostic analysis (Figure 3A). We further validated the results in the GEPIA and BloodSpot database (Figures 3B,C). We found higher expression of CAVIN2 was certainly associated with a poorer prognosis (*p* is equal to 0.037 and 0.0022 individually), but the expression levels of other members have no statistical correlation with the prognosis of leukemia.

### Cavin Family Members Are Under-Expressing in Primary Cells of Children With Acute Leukemia, Especially in Myeloid Leukemia

**Figure 4:**
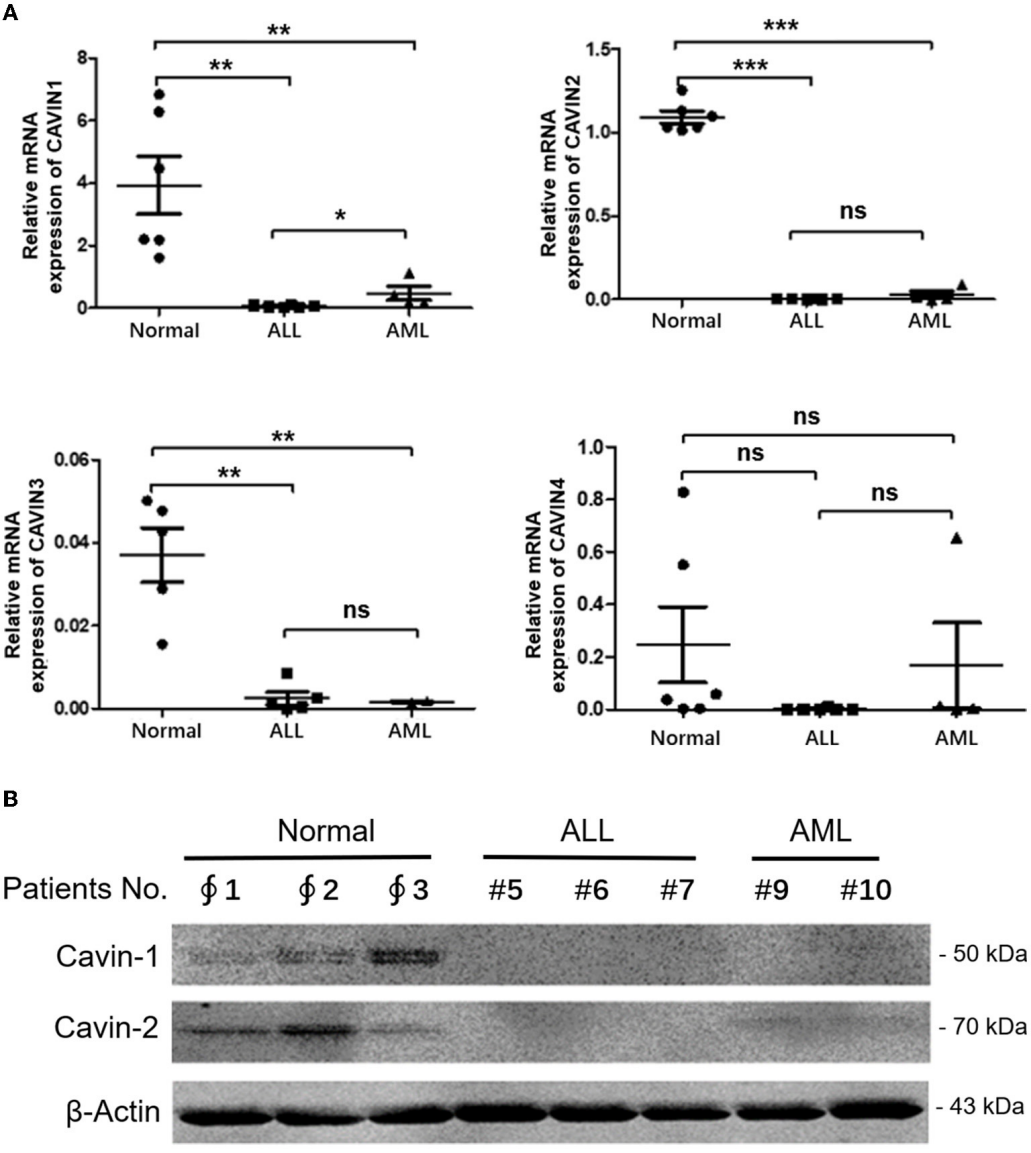
The transcription levels of Cavin family members in leukemia. **(A)** After mononuclear cells isolated and RNA extracted from peripheral blood or bone marrow of patients or health donars, the RNA levels of Cavin family members were analyzed by reverse transcription-quantitative polymerase chain reaction (qRT-PCR). Bar graphs: mean ± SEM; *, *p* < 0.05; **, *p* < 0.01; ***, *p* < 0.001, one-way ANOVA, *post-hoc* comparisons, Dunnett's test. **(B)** Mononuclear cells were isolated from healthy donors and patients with leukemia. Cytoplasmic and nuclear extracts were analyzed by Western blot using specific antibodies for Cavin-1, Cavin-2 and β-Actin. ∮ Normal donors; # Leukemia patients.

Finally, the mRNA expression levels of Cavin family members in leukemia patient samples were further detected via qRT-PCR. The results show that the transcription of CAVIN1, CAVIN2 and CAVIN3 were significantly lower than in normal samples from healthy donors, and the expression of CAVIN1 in myeloid leukemia is significantly higher than that in lymphocytic leukemia (*p* < 0.05) (Figure 4A). In addition, we verified the Cavin-1 and Cavin-2 protein levels by Western blotting and indicated they were also lower in leukemia cells than in normal peripheral blood mononuclear cells (PBMCs) (Figure 4B).

## Discussion

Recently, Cavin family members were found to be downregulated in multiple cancers and their tumor suppression properties have been confirmed via *in vitro* and *in vivo* assays (22, 23). Cavin-1 and Cavin-3 were down-regulated in breast cancer with a low progression-free survival rate (6). However, the expression level of the Cavin family in leukemia and the relationship between expression and prognosis are still not known.

In the attempt to understand the expression of the Cavin family in childhood leukemia and its implications in subtype diagnosis and prognosis. In this study, we performed a comprehensive bioinformatics analysis and validation experiments (Figure 1A), and found that the four Cavin members were lowly expressed in leukemia, especially in lymphoblastic leukemia. The expression of CAVIN1 and CAVIN2 is higher in myeloid leukemia than lymphoblastic leukemia, while CAVIN4 expression is just the opposite in leukemia. CAVIN1 and CAVIN2 complement each other with a significant linear positive correlation. CAVIN2 relatively high expression was often found in myeloid leukemia and predicted a poorer prognosis, which may be regarded as potential prognostic biomarkers, diagnostic subtype indicators and therapy targets for leukemia.

In our study, ONCOMINE and GEPIA dataset analysis revealed that the mRNA expression of Cavin family members, especially CAVIN1 and CAVIN2, was lower in human leukemia than that in normal cells, as the same as in most tumors (Figures 1B,C). Moreover, we further evaluated the expression of these four members in diverse types of human leukemia cell lines via the CCLE and BloodSpot database. As shown in Figures 1D,E, all Cavin family members were significantly downregulated in lymphoblastic leukemia except for CAVIN4. CAVIN1 and CAVIN2 are higher expressed in myeloid leukemias (including AML and CML) than that in lymphocytic leukemias (including ALL and CLL). CAVIN3 can be either overexpressed or underexpressed in myeloid leukemias and lymphoblastic leukemia. However, compared with lymphocytic leukemia, CAVIN4 expression is relatively higher in lymphocytic leukemia than that in myeloid leukemia, especially in acute lymphoblastic leukemia. Then we analyzed the correlation between Cavin family member pairs in Leukemia by LinkedOmics and GEPIA database. We found the mRNA expression levels of CAVIN1 and CAVIN2 complement each other with a significant positive correlation (R is 0.3425 and 0.47 individually) (Figure 2).

To investigate the inner linkage between the phenotype and prognosis of leukemia, we also analyzed the prognostic value of the mRNA levels of Cavin family members in patients with leukemia by the R programming language and data from LinkedOmics. In particular, overexpressed CAVIN4 was associated with a better overall survival (OS), as we expected, but there was obviously better OS with high vs. low expression of CAVIN2 in leukemia (*p* = 0.017) by R-programing in our report (Figure 3A). We then used the GEPIA and BloodSpot database to validate the unexpected result and found that high CAVIN2 expression was significantly associated with poorer OS in leukemia (*p* is 0.037 and 0.022 individually), but the trend was not significant for CAVIN1, CAVIN3 and CAVIN4 (Figures 3B,C).

In order to explain this strange phenomenon, we reviewed the relevant literature. Cavin-2 is critical organizer of caveolae and plays a critical role in tumor cell proliferation, migration and invasion (22). Cavin-2 downregulation in cancer tissues vs. control tissues has been reported in several malignant tumors, such as oral squamous cell carcinoma, and is associated with tumor progression (24). Cavin-2 is an essential factor for angiogenesis by increasing the production of nitric oxide (NO) in endothelial cells (15). However, ectopic Cavin-1 overexpression inhibits tumor growth and metastasis due to antiangiogenesis- and antilymphangiogenesis-regulating functions as is reported in prostate cancer (25). Several studies show that high levels of Cavin-2 facilitate caveolin-1 recruitment and colocalization. Caveolin-1 is the major essential coat protein for caveolae formation and is implicated in tumoral growth and angiogenesis. Caveolin-1 is expressed at high levels in leukemia cells, such as AML, CML, adult T-cell leukemia (ATL) and chronic lymphocytic leukemia (CLL) cells, compared with normal PBMCs (26–29). Previous studies have shown that caveolin-1 and multidrug resistance-1 (MDR-1) gene expression levels are positively correlated in relapsed leukemia (30). MDR-1 produces the membrane protein P-glycoprotein (P-gp) and acts as an efflux pump for cytotoxic drugs (30). It has been shown that P-gp is localized to caveolae and coimmunoprecipitates with caveolin-1 (30). Moreover, the coordinated overexpression of Cavin-2 and caveolin-1 leads to compartmentation into caveolae, which prevents epidermal growth factor receptor (EGFR) degradation and simultaneously enables intracellular EGFR kinase-linked signaling (28, 29). All in all, higher Cavin-2 may promote the resistance and progression of leukemia by regulating cell differentiation, proliferation, angiogenesis and MDR-1 or EGFR expression. As we know, the prognosis of ALL is better than that of AML, and the prognosis of B-ALL is generally better than that of T-ALL. In the present study, altered expression of Cavin family members, especially high mRNA levels of CAVIN2 and low levels of CAVIN4, indicating a poor outcome, were found in AML, CML and T-ALL, as shown in Figure 1D. We might hypothesize that the Cavin family, especially CAVIN2 and CAVIN4, could be potential markers of the clinicopathological parameters of patients with different types of leukemia.

However, there are some limitations of the present study. On the one hand, the sample size was small, and all patients were from two clinical pediatric centers, which means it is hard to eliminate selection bias. On the other hand, the biological functions and resistance mechanisms of the Cavin family in leukemia remain unknown. Therefore, additional investigations will be needed to investigate this issue.

## Data Availability Statement

Raw data of published western blot and gene expression arrays of all samples are available in additional accessory files (Supplementary Material). And the 5 datasets used in this study are: GEPIA (http://gepia.cancer-pku.cn); ONCOMINE (https://www.oncomine.org/); CCLE (https://www.broadinstitute.org/ccle); LinkedOmics (http://www.linkedomics.org/login.php); and BloodSpot (http://servers.binf.ku.dk/bloodspot/). Any additional information can be provided upon request.

## Ethics Statement

The studies involving human participants were reviewed and approved by the Sun Yat-sen University Ethics Committee. Written informed consent to participate in this study was provided by the participants' legal guardian/next of kin.

## Author Contributions

JY and JH designed the research. JY, YT, CL, and HW collected the data. JH and YT finished the qRT-PCR and Western blotting. JY, JH, and HW conducted the statistical analysis. JY, QZ, and JH wrote the manuscript. JH and CC contributed to the review and editing of the manuscript. CC acquired the funding to support our research. All authors have read and approved the manuscript.

## Funding

This research was funded by the Sanming Project of Medicine in Shenzhen (SZSM202011004) and the National Natural Science Foundation of China (8157010694).

## Conflict of Interest

The authors declare that the research was conducted in the absence of any commercial or financial relationships that could be construed as a potential conflict of interest.

## Publisher's Note

All claims expressed in this article are solely those of the authors and do not necessarily represent those of their affiliated organizations, or those of the publisher, the editors and the reviewers. Any product that may be evaluated in this article, or claim that may be made by its manufacturer, is not guaranteed or endorsed by the publisher.

## Abbreviations

AML: acute myeloid leukemia
ATL: adult T-cell leukemia
ALL: Acute lymphoblastic leukemia
CART: chimeric antigen receptor T cells
CLL: chronic lymphocytic leukemia
CCLE: Cancer Cell Line Encyclopedia
CGL: congenital generalized lipodystrophy
EGFR: epidermal growth factor receptor
MDR-1: multidrug resistance-1MMP-9 matrix metalloproteinase 9
MURC: muscle-related skeletal and cardiac
OS: overall survival
PBMCs: peripheral blood mononuclear cells
P-gp: P-glycoprotein
PTRF: polymerase 1 transcript release factor
PRKCDBP: protein kinase c delta binding protein
qRT-PCR: quantitative real-time Polymerase Chain Reaction
RIPA: radioimmunoprecipitation assay
SDPR: serum deprivation response protein
PKC: Protein Kinase C.
