# Supplementary material for: Expression of the Cavin Family in Childhood Leukemia and Its Implications in Subtype Diagnosis and Prognosis Evaluation
Source: Front Pediatr. 2022 Jun 3;10:815421. doi: 10.3389/fped.2022.815421 (PMC9203855; doi:10.3389/fped.2022.815421)
Supplement: Supplementary file 2 [file Image_1.pdf]

Title

Expression of the Cavin family in childhood leukemia and its implications in subtype diagnosis and prognosis evaluation

Jing Yang[1†], Junbin Huang[1†], Yong Liu[1], yanlai Tang[2], Chao Lin[1], Huabin Wang[1], Qin Zhou[1], Chun Chen[1*]

[1] Division of Hematology/Oncology, Department of Pediatrics, The seventh affiliated hospital, Sun Yat-Sen University, 628 Zhenyuan Road, Guangming, Shenzhen, Guangdong, 518107, P.R. China.

[2] Department of Pediatrics, The First Affiliated Hospital, Sun Yat-sen University, Guangzhou 510080, Guangdong Province, P. R. China

†These authors contributed equally to this study.

* Corresponding author. E-mail addresses: chenchun69@126.com.

Supplementary Table 1 The data was included in an Excel file as another accessory.

Supplementary Figure 1

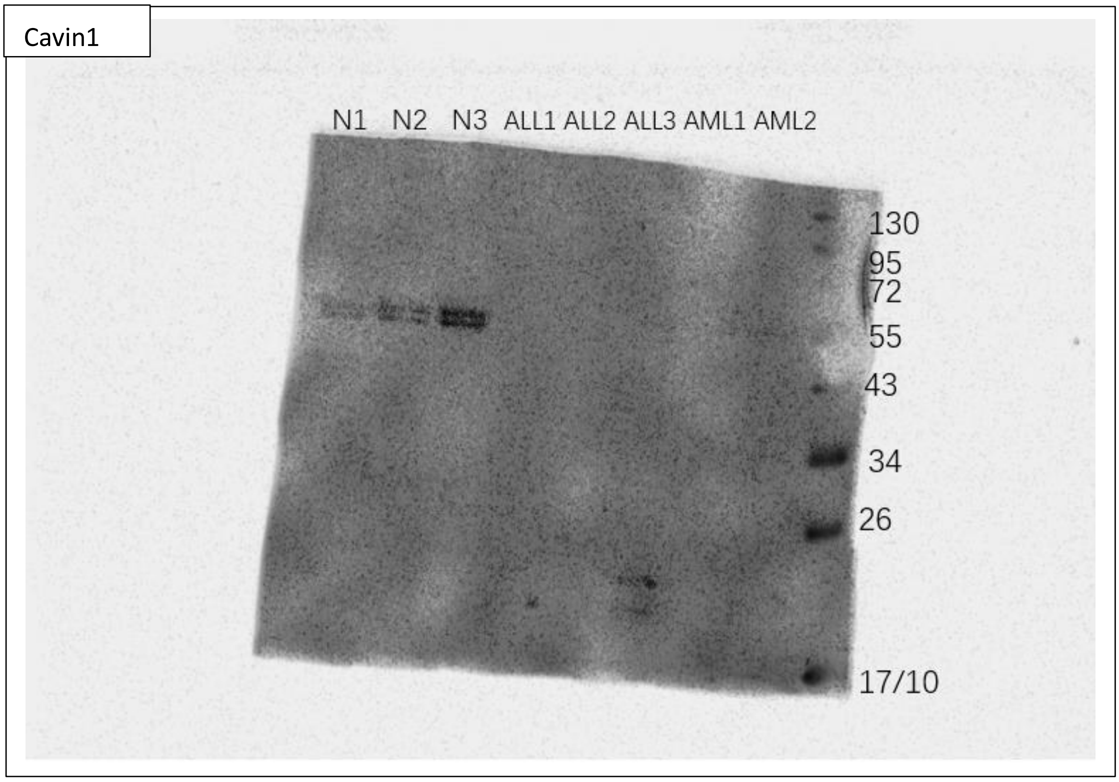
Cavin1
N1 N2 N3 ALL1 ALL2 ALL3 AML1 AML2
130
95
72
55
43
34
26
17/10

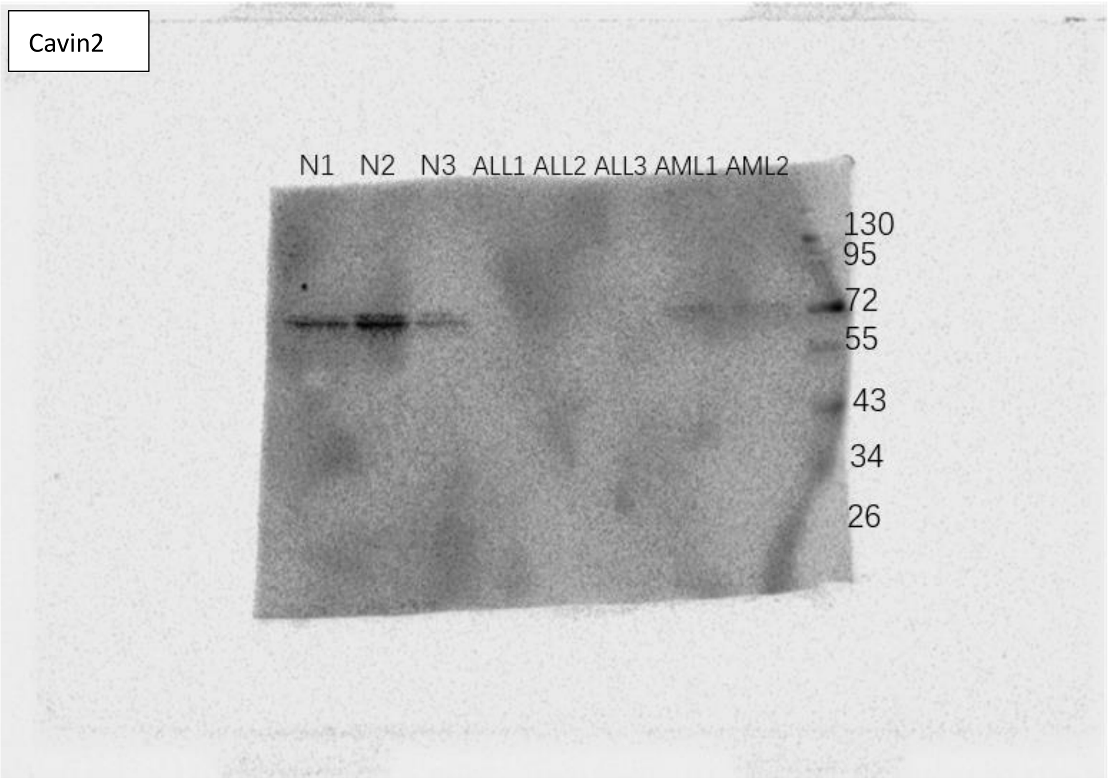
Cavin2
N1 N2 N3 ALL1 ALL2 ALL3 AML1 AML2
130
95
72
55
43
34
26

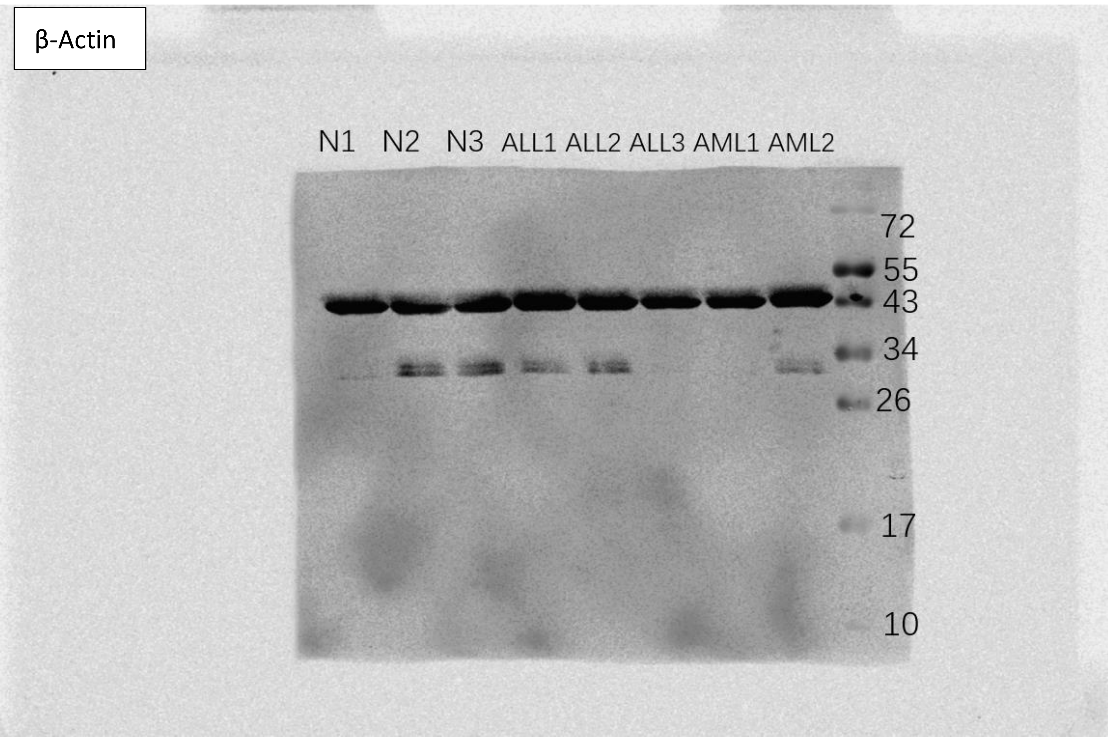
β-Actin
N1
N2
N3
ALL1
ALL2
ALL3
AML1
AML2
72
55
43
34
26
17
10
